# Quantum dot-enabled infrared hyperspectral imaging with single-pixel detection

**DOI:** 10.1038/s41377-024-01476-4

**Published:** 2024-05-28

**Authors:** Heyan Meng, Yuan Gao, Xuhong Wang, Xianye Li, Lili Wang, Xian Zhao, Baoqing Sun

**Affiliations:** 1 School of Information Sciences and Engineering, Shandong University, Qingdao, China; 2 Center for Optics Research and Engineering (CORE), Key Laboratory of Laser & Infrared System (Shandong University), Ministry of Education, Shandong University, Qingdao, China; 3 School of Mechanical, Electrical and Information Engineering, Shandong University, Weihai, China

**Keywords:** Quantum dots, Imaging and sensing, Nanophotonics and plasmonics

## Abstract

Near-infrared (NIR) hyperspectral imaging is a powerful technique that enables the capture of three-dimensional (3D) spectra-spatial information within the NIR spectral range, offering a wide array of applications. However, the high cost associated with InGaAs focal plane array (FPA) has impeded the widespread adoption of NIR hyperspectral imaging. Addressing this challenge, in this study, we adopt an alternative approach—single-pixel detection for NIR hyperspectral imaging. Our investigation reveals that single-pixel detection outperforms conventional FPA, delivering a superior signal-to-noise ratio (SNR) for both spectral and imaging reconstruction. To implement this strategy, we leverage self-assembled colloidal quantum dots (CQDs) and a digital micromirror device (DMD) for NIR spectral and spatial information multiplexing, complemented by single-pixel detection for simultaneous spectral and image reconstruction. Our experimental results demonstrate successful NIR hyperspectral imaging with a detection window about 600 nm and an average spectral resolution of 8.6 nm with a pixel resolution of 128 × 128. The resulting spectral and spatial data align well with reference instruments, which validates the effectiveness of our approach. By circumventing the need for expensive and bulky FPA and wavelength selection components, our solution shows promise in advancing affordable and accessible NIR hyperspectral imaging technologies, thereby expanding the range of potential applications.

## Abstract (web summary)

We implement single-pixel detection alongside colloidal quantum dot (CQD) light filters to enable low-cost and effective near-infrared (NIR) hyperspectral imaging.

## Graphical abstract

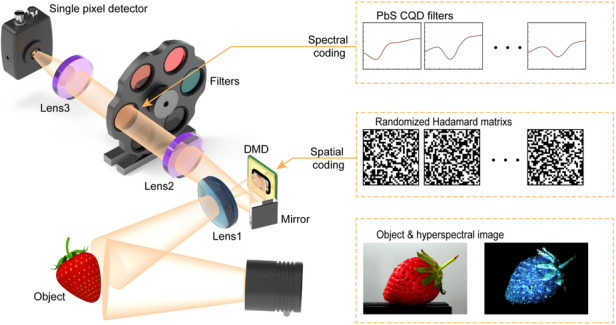

## Introduction

Near-infrared (NIR) hyperspectral imaging provides detailed spectral information across a wide range of wavelengths in the near-infrared region, typically from approximately 780 nm to 2500 nm^1,2^. This enables the identification and characterization of materials and chemical compounds based on their spectral signatures, which can be used for various applications such as chemical analysis^3,4^, material identification^5,6^, and quality control^7,8^. To resolve the spectral information of a scene, scientists have employed various strategies, including dispersive optics, narrow-band light filters, and interferometric architectures^9^. However, each of these approaches has its limitations. Dispersive optics can improve spectral resolution by increasing the groove density and focal length^10^. However, this approach also increases the complexity of fabrication and the overall size of the device. In addition, the spectral resolving techniques using interferometry rely on controlling the optical path difference with subwavelength precision^11^. However, they are sensitive to vibrations and motions, and their spectral measurement window is limited by the optical path modulation range. Hyperspectral imaging systems that utilize narrow-band filters often encounter a compromise between spectral and spatial resolution^12^. In order to achieve higher spectral resolution, more filters are typically required. However, this trade-off results in an increased number of “super-pixels” composed of fewer individual pixels.

With advancements in algorithms and computational capabilities, computational spectral reconstruction based on broad-band light encoding has gained attention^9^. Techniques such as metasurfaces and photonic crystal structures have been explored^13–16^. However, these architectures may suffer from optical quenching losses and have limitations in the angular detection range^17^. One promising approach is the use of colloidal quantum dots (CQDs)^18,19^, which can tune their absorption characteristics continuously from ultraviolet to mid-infrared by adjusting their size and chemical composition. Therefore, CQDs offer a convenient solution for fabricating broad-band filters.

Hyperspectral images can be represented as a three-dimensional (3D) (*x*, *y*, *λ*) data cube for processing and analysis, where *x* and *y* represent two spatial dimensions of the scene, and *λ* represents the spectral dimension (comprising a range of wavelengths). The hyperspectral cube is typically obtained via spatial or spectral scanning^20–22^. Therefore, for NIR hyperspectral imaging, a two-dimensional (2D) infrared sensor is required to capture the spatial (*x*, *y*) map of the scene or a full slit spectrum (*x*, *λ*) for spectral or spatial scanning, respectively. However, fabricating large-scale InGaAs detector arrays with high yield can be challenging due to the complex fabrication processes involved and the sensitivity of the detectors to process variations^23–26^. Integrating the 2D InGaAs detector arrays into practical imaging systems often requires complex packaging and integration processes^27–29^. Challenges include the need for hermetic sealing to protect the detectors from environmental factors, proper alignment and bonding of the detectors to readout electronics, and optimizing the optical coupling between the detectors and the imaging optics^30–32^. Therefore, 2D NIR detector arrays are typically produced in lower volumes and yields compared to visible light cameras. As a result, they are frequently employed in a constrained range of applications, including scientific research, military purposes^33^, industrial inspection, and medical imaging^34,35^.

To avoid the expensive 2D NIR sensors and complex wavelength selection components, making the NIR hyperspectral imager more affordable for daily usages, we propose using single-pixel detection in conjunction with CQD filters. In this study, we reveal a notable enhancement in signal-to-noise ratio (SNR) for both spectral and image reconstruction through single-pixel detection, surpassing the performance of array detector. The encoding of spectral information employs PbS CQD filters, offering a scalable solution with cost-effective and versatile solution-processing capabilities. By varying the size of the CQDs, the transmission characteristics of the CQD filters are changed and thereby the spectral information of the signal is modulated^19,36–38^. Meanwhile, by varying the pattern of a digital mirror device (DMD), we are able to encode the spatial information of the scene. Therefore, the signals that are received by the single-pixel InGaAs detector have been modulated spectrally and spatially by the CQD filters and the DMD. As a result, using the compressed sensing algorithm, we can obtain the NIR hyperspectral image with a picture resolution of 128 × 128, where each pixel contains a spectral signature (from 1050 to 1630 nm), by correlating the signals with the transmission spectra of the CQD filters and the patterns generated using DMD.

## Results

### Assessing noise Resilience in hyperspectral imaging: single-pixel vs. focal plane array (FPA) detection

**Fig. 1:**
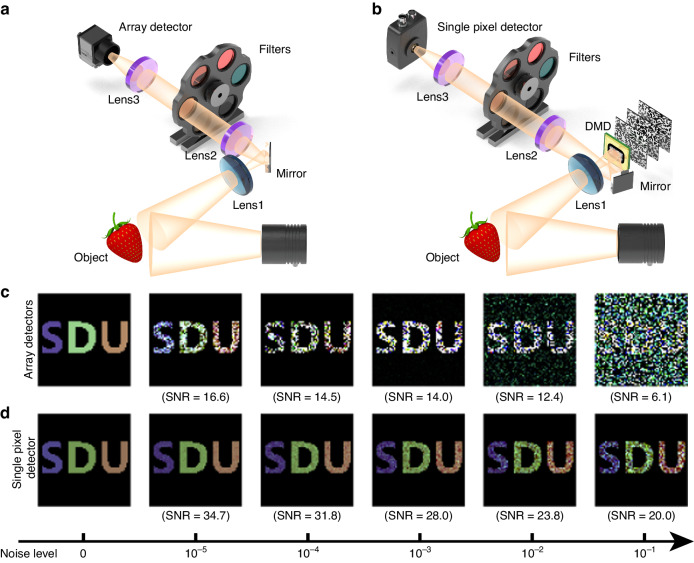
Comparison between CQD-based hyperspectral imaging using FPA and single-pixel detection. Schematic diagram of the NIR hyperspectral imaging system using **a** FPA and **b** a single-pixel detector. Simulated hyperspectral images under increasing levels of noise using **c** FPA and **d** a single-pixel detector

Unlike the use of conventional NIR FPA (Fig. 1a), our hyperspectral imaging system employs single-pixel detection, as depicted in Fig. 1b. Illumination of the object is achieved using a tungsten-halogen lamp, emitting white light with an infrared spectral component spanning wavelengths from 200 to 3000 nm. The lens images the object onto the DMD. Utilizing the DMD and CQD filters, we encode both the spatial and spectral characteristics of the object. The overall signals are recorded through a single-pixel InGaAs detector. By correlating the recorded light intensity with the spectral and spatial modulation achieved by the CQD filters and DMD, we can efficiently reconstruct the object’s image and obtain spectral information for each pixel within the image. Our NIR hyperspectral imaging system allows for the capture of spatially resolved images while offering comprehensive spectral details.

To demonstrate the superior performance of single-pixel detection over FPA, a series of simulations were conducted to assess the noise tolerance of hyperspectral imaging system using FPA (Fig. 1a) and single-pixel detector (Fig. 1b) for spectral and image reconstruction, employing mean square deviation as the evaluation metric. As illustrated in Fig. S1, these simulations involved imaging the letters S, D, and U, each characterized by distinct spectral curves (represented by three different colors in Fig. S1), as the simulation targets. Hyperspectral imaging was executed employing both FPA and a single-pixel detector. Given that dark noise serves as the primary source of detection noise in linear photoelectric detection and approximately follows a Gaussian distribution, a Gaussian-like random distribution was adopted as the noise model for the simulations. The image results and theoretical analysis of the simulations are shown in Supplementary Information (Notes 1 and 2).

In Fig. 1c, d, the hyperspectral imaging results of the letters “SDU” utilizing array detectors are depicted. Throughout the imaging process, noise independently affects the output signals of each individual pixel in the FPA, leading to a degradation in both spatial and spectral information reconstruction quality. As a result, as noise levels increase, the quality of the captured hyperspectral images using FPA notably deteriorates. Conversely, with the single-pixel detection approach, noise levels impact the collective response of all pixels. Through correlation across all pixels, the influence of noise on both image and spectral reconstruction is mitigated (see Eq. S4 in the Supplementary Information). Consequently, as shown in Figs. S2 and S3, although the quality of hyperspectral imaging achieved using the single-pixel detector slightly diminishes with increasing noise levels, the overall quality is significantly superior to that obtained using FPA under the same noise level. Therefore, by comparing these two approaches in terms of image and spectral reconstruction quality, it can be concluded that employing single-pixel detection for hyperspectral imaging offers enhanced resistance to noise.

### Computational methods for hyperspectral image reconstruction

We employ computational methods to reconstruct both the spectral and spatial information of the image as shown below:

1 $$Y=F\left(\lambda \right)T\left(\lambda ,r\right)H\left(r\right)$$

To simplify the model, we transform the 3D hyperspectral data cube of target into a matrix *T*(*λ*, *r*). $F(\lambda )$ represents the spectral modulation matrix for the target, $H\left(r\right)$ represents the spatial modulation matrix, and $Y$ represents the measurement output of the single-pixel detector.

When the object $T\left(\lambda ,r\right)$ is modulated with the *i*th $(i=1,\,2,\,3,\,\ldots ,{m})$ CQD color filter ${F}_{i}\left(\lambda \right)$ and *j*th $(j=1,\,2,\,3,\,\ldots ,{n})$ DMD pattern ${H}_{j}\left(r\right)$, the resulting detected light intensity ${y}_{i,j}$ can be written as

2 $${y}_{i,j}=\sum _{\lambda }\left\{{F}_{i}\left(\lambda \right)\sum _{r}\left[T\left(\lambda ,r\right){H}_{j}\left(r\right)\right]\right\}$$

Through conducting *m* × *n* measurements, we can reconstruct both the spectra and image of the object. To achieve this, we employed generalized alternating projection based total variation (Gap-TV), a compressed sensing algorithm, for spectral reconstruction. In order to enhance the quality of the reconstructed image, we utilized an orthogonal Hadamard matrix as the spatial modulation matrix and ensured that the target was fully sampled. Details of this algorithm can be found in Supplementary Information (Note 3).

**Fig. 2:**
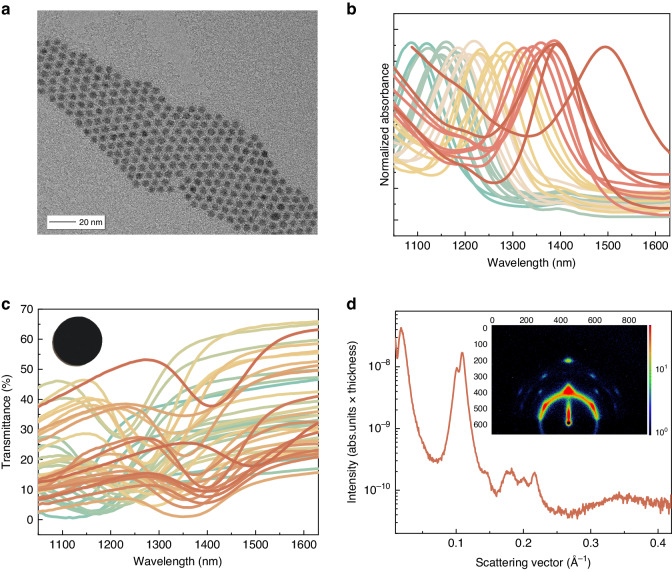
Characterization of the PbS CQD filters. **a** HRTEM image of PbS CQDs. **b** Absorption spectra of PbS CQDs in solution. **c** Transmission spectra of PbS CQD light filters fabricated by drop-casting. The inset shows a photograph of the PbS CQD light filter. **d** SAXS pattern and corresponding 1D profile of the drop-cast PbS CQD film

We synthesized a series of PbS CQDs with different sizes by carefully controlling the sulfide precursor injection temperature and the amount of oleic acid^39^. Figure 2a illustrates a representative high-resolution transmission electron microscopic (HRTEM) image of the PbS CQDs, demonstrating their excellent size monodispersity. The absorption spectra of the PbS CQDs dissolved in tetrachloroethene solution are presented in Fig. 2b, exhibiting a significant peak-to-valley ratio ranging from 0.5 to 1, indicative of the uniformity of the CQDs. To fabricate CQD filters, we dropcast a solution of PbS CQDs (10% v v^−1^ octane in hexane) onto glass substrates and allowed the solution to evaporate slowly. The resulting transmission spectra of the PbS CQD filters are shown in Fig. 2c, with the inset demonstrating the uniform CQD film obtained on the glass substrate. Moreover, Fig. 2d displays the small-angle X-ray scattering (SAXS) pattern of an as-fabricated CQD filter. The pattern reveals the self-assembly of the majority of PbS CQDs into a superlattice structure upon solvent evaporation. The formation of these superlattices indicates the high monodispersity of our CQDs and contributes to the improved absorption capability of the filter through enhanced dipole coupling^40–42^.

To obtain spectral information from a target, it is necessary to encode and modulate the incident light field in a spectrally controlled manner. One effective approach is to modify the bandgap of a material, which in turn alters its absorption edge and allows for the creation of a series of absorptive color filters. By adjusting the material’s chemical composition, a set of edge-pass filters with distinct step-like absorption profiles can be achieved. In the case of CQDs, their absorption properties can be precisely tuned by manipulating their sizes. Due to the presence of excitonic peaks, quantum dots exhibit absorption characteristics with intricate and detailed features. Here we compare the spectral encoding capabilities of CQD filters with those of edge-pass filters achieved through bandgap engineering.

### Evaluating spectral reconstruction performance: CQD filters vs conventional edge-pass and band-pass filters

**Fig. 3:**
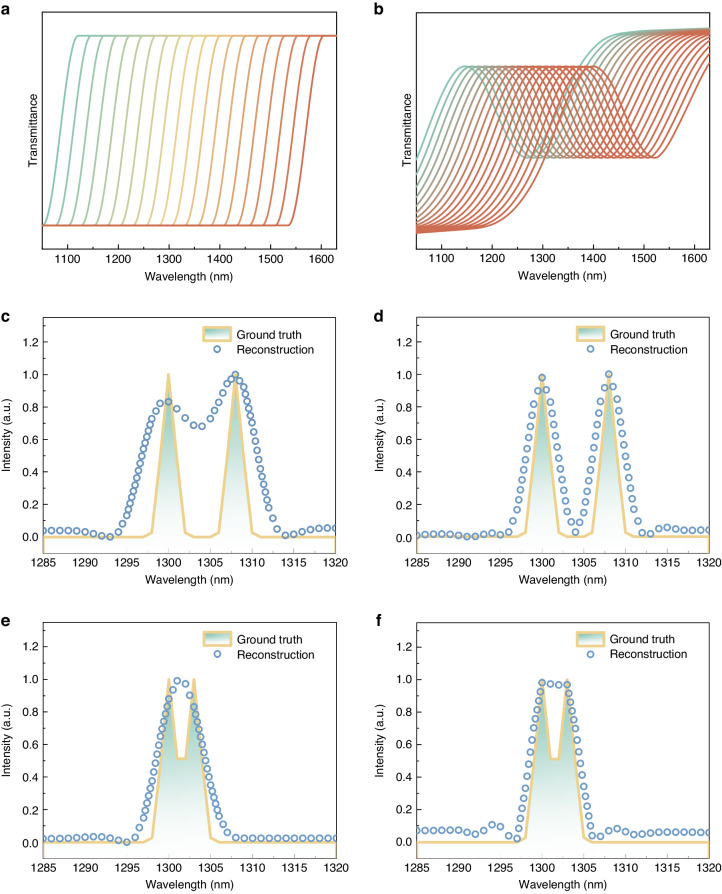
A comparison of the spectral resolving capabilities between step-like edge-pass filters and CQD light filters. The typical transmission spectra of **a** step-like edge-pass filters and **b** CQD filters are compared. In addition, the spectra are reconstructed using 100 **c**, **e** CQD filters, as well as **d**, **f** step-like edge filters

To showcase the disparity in spectral resolving capabilities between step-like edge-pass filters and CQD filters, we utilized both types of filters to encode and reconstruct a pair of Gaussian peaks with a Full Width at Half Maximum (FWHM) of 1 nm and varying separation distances^19^. The comparison of spectral reconstruction results obtained using these filters is presented in Fig. 3c–f. With 100 edge-pass filters, the minimum distinguishable wavelength separation is 6 nm. In contrast, employing an equal number of CQD filters with excitonic peaks allows for the discrimination of wavelengths with a minimum separation of 3 nm. The substantial improvement in spectral reconstruction resolution using CQD filters can be attributed to the following reasons. Step-like edge-pass filters utilize binary encoding by switching transmission on and off at specific wavelengths, limiting their spectral resolution capabilities and inability to distinguish spectral information above or below the absorption edge^43,44^. On the contrary, due to the randomness and variability present in the absorption spectra of CQDs, CQD filters facilitate precise modulation of target spectra across various wavelengths, thereby enabling finer spectral resolution and yielding improved reconstruction outcomes. In addition, edge-pass filters often exhibit significant overlap in their transmission spectral curves, reducing correlation efficiency. CQD filters offer a better solution by precisely tuning the optical absorption characteristics through CQD size control, resulting in distinct and less correlated filter responses. Furthermore, edge-pass filters can lead to invalid sampling due to element correlation in the measurement matrix, increasing reconstruction time and impacting system performance. The measurement matrix composed of edge-pass filter transmission spectra has a high condition number, making it ill-conditioned and susceptible to noise, negatively affecting spectral reconstruction. On the other hand, the measurement matrix composed of CQD filter transmission spectra has a low condition number, providing greater resilience to noise and improved system stability. We conducted a similar comparison using band-pass filters. Consistently, we found that CQD filters enhance spectral reconstruction capabilities. For further details, please refer to Note 4 in the Supplementary Information. Thus, CQD filters offer more effective spectral sampling and reconstruction capabilities compared to edge-pass and band-pass filters, establishing them as the preferred choice for spectral modulation.

**Fig. 4:**
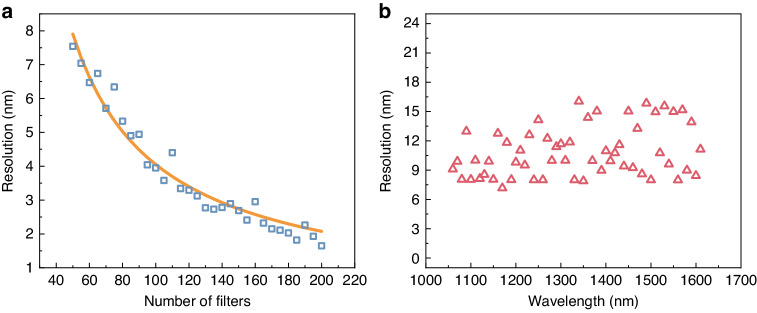
Characterization of spectral resolution. **a** Relationship between spectral resolution and the number of CQD filters at 1300 nm. **b** Spectral resolution across the measurement window at different wavelengths using 50 filters

To investigate the relationship between spectral resolution and the number of PbS CQD filters used in spectral reconstruction, we conducted a numerical simulation. We utilized a Gaussian signal centered at 1300 nm with an FWHM of 1 nm as our target and reconstructed it using different numbers of filters^18^. The FWHMs of the reconstructed curves represented the spectral resolution of the system for each filter quantity. As shown in Fig. 4a, increasing the number of color filters improved the spectral resolution, although the incremental enhancement gradually diminished with each additional filter. To validate the simulation findings, we conducted spectral reconstruction experiments using 10, 20, 30, 40, and 50 CQD filters. Our experimental results aligned consistently with the outcomes of the simulations (see Supplementary Information Note 5). For our proof-of-concept experiment, we chose 50 color filters for convenience, achieving a spectral resolution of 7.89 nm at 1350 nm, equivalent to an energy resolution of 5.37 meV. We evaluated the spectral resolving capability of our system across the entire measurement range (from 1050 to 1630 nm) using the 50 CQD filters we developed, as illustrated in Fig. 4b. The system demonstrated its highest resolution at 1170 nm, with a spectral resolution of 7.16 nm. On average, the resolution across the measurement range was 8.59 nm.

### Hyperspectral imaging of emissive, transmissive, and reflective objects

**Fig. 5:**
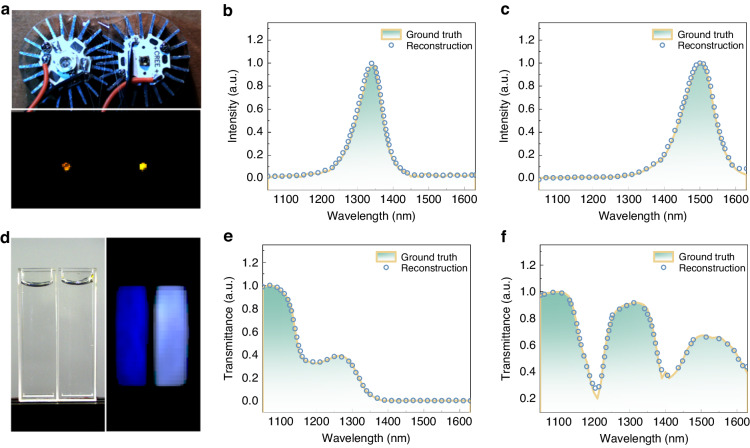
Hyperspectral images of emissive and transmissive. **a** Color photo and hyperspectral image of two switched-on NIR LEDs with wavelengths of 1350 nm and 1500 nm. **b**, **c** Comparison of the emission spectra of LEDs measured using a commercially available spectrometer and extracted from the hyperspectral image. **d** Color photo and hyperspectral image of two transparent solutions (left: water, right: oleic acid) in cuvettes. **e**, **f** Comparison of the transmission spectra of the two solutions measured using a commercially available spectrometer and extracted from the hyperspectral image

To evaluate the spectral reconstruction capabilities of our system, we measured the emission spectra of two NIR LEDs (light-emitting diodes) with peaks at 1350 nm and 1500 nm. We reconstructed the hyperspectral image of the LEDs and applied pseudocolor based on wavelength (see Note 6 in Supplementary Information). As shown in the lower panel of Fig. 5a, the left LED emitted at a longer wavelength compared to the right LED. Extracting the spectral information from both LEDs, the reconstructed spectra (dotted curves, Fig. 5b, c) closely matched those obtained from commercially available spectrometers.

NIR hyperspectral imaging provides spatial and spectral information in a single image, enabling simultaneous acquisition of both data types. This capability facilitates the identification and characterization of materials or chemical compounds with spatial distribution, offering insights into sample heterogeneity and spatial patterns. In the left panel of Fig. 5d, naked eyes cannot differentiate between water and oleic acid since both solutions are colorless and transparent in visible light. However, through hyperspectral imaging, we captured an image of two cuvettes and observed distinct absorption behaviors of water and oleic acid. This allowed easy identification of the liquid in each cuvette. The transmission spectrum of water (left cuvette) is shown in Fig. 5e, with a dip at 1180 nm attributed to a vibrational overtone of the O–H bond, and absorption near 1430 nm due to the first overtone of O–H stretching. For oleic acid (Fig. 5f), the valley at 1210 nm corresponds to the second overtone of C–H stretching, while the valley at 1430 nm is associated with the first overtone of O–H stretching^45^.

**Fig. 6:**
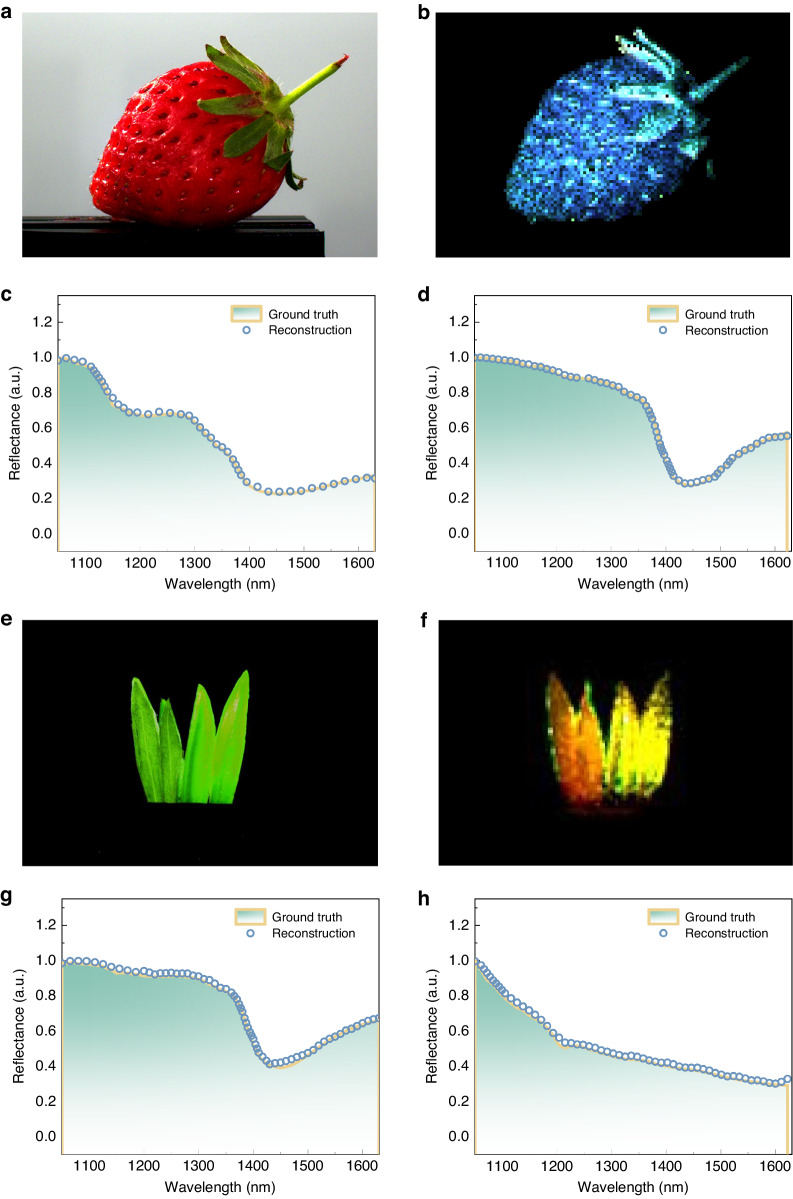
Hyperspectral images of reflective objects. **a** Color photo and **b** hyperspectral image of a fresh strawberry. The reconstructed reflection spectra of the **c** receptacle (flesh) and **d** sepal (leaf) of the strawberry. **e** Color photo and **f** hyperspectral image of a mixture of real and plastic grasses. The reconstructed reflection spectra of the **g** real grasses and **h** plastic grasses

NIR hyperspectral imaging offers non-destructive imaging of samples, making it suitable for applications where sample integrity is critical, such as in pharmaceuticals, food processing, and cultural heritage preservation. In our study, we demonstrate hyperspectral imaging of fresh and delicate strawberries. Figures 6a, b are visible color photo and hyperspectral image of a fresh strawberry. The receptacle (flesh), sepal (leaf), and seeds of the strawberry shows distinct spectral response (Fig. 6b). As shown in Fig. 6c, d, the reflection spectra of the receptacle and sepal closely match those measured with a commercial spectrometer. The reflection spectrum of the receptacle (Fig. 6d) resembles that of water, indicating a high water content. We also captured a hyperspectral image of a tangerine (Supplementary Information, Fig. S9). The CQD hyperspectral imager provides clear images of the fruits and spectral information about different parts without damage, allowing quick identification and characterization of samples while reducing the need for expensive and time-consuming laboratory analysis. This technology has the potential to significantly improve product quality control, including evaluating sugar or water content in fruits or crops.

In the context of camouflage recognition, hyperspectral imaging plays a critical role in detecting and identifying concealed objects, patterns, or materials that may be challenging to distinguish with the naked eye or traditional imaging methods. Figure 6e demonstrates a scenario where a combination of real and plastic grasses cannot be differentiated visually. However, through hyperspectral imaging, which we have implemented, the fake and real grasses exhibit distinct NIR reflection characteristics, as depicted in Fig. 6f. The reflection spectra of the real and fake grasses are illustrated in Fig. 6g, h, respectively. In Fig. 6g, it is evident that the real grasses display an absorption dip around 1400 nm, which corresponds to the absorption of O–H bonds in actual vegetation. Consequently, by utilizing our NIR hyperspectral imaging system, we are capable of discerning counterfeit plants from genuine vegetation.

## Discussion

In summary, our study presents a NIR hyperspectral imager that employs CQD filters and single-pixel detection. We demonstrate that single-pixel detection offers superior noise tolerance compared to conventional FPA, while CQD filters enhance spectral resolution in contrast to edge-pass filters. By utilizing 50 self-assembled CQD filters, we successfully achieved spectral reconstruction across a broad wavelength range of 1050 nm to 1630 nm, with an average spectral resolution of 8.59 nm. The resulting hyperspectral image offers a resolution of 128 × 128 pixels, with each pixel providing a complete spectrum.

Significantly, the reconstructed spectra exhibit excellent consistency with commercially available spectrometers, confirming the accuracy and reliability of our approach. By leveraging the flexible fabrication process of CQDs and advancements in optical field modulation techniques, our system has the potential to miniaturize both the spectral and spatial modulation modules, ultimately leading to the miniaturization of the entire hyperspectral imaging system. By combining a single-pixel detector with CQD filters, we eliminate the need for a costly 2D arrayed sensor typically employed in conventional hyperspectral imaging systems, thereby reducing system complexity and cost. The attained spectral reconstruction and spatial resolving capabilities showcase the effectiveness of our system and the promising potential for affordable and portable hyperspectral imaging devices. Moreover, our strategy integrates both spectral and spatial encoding, potentially allowing for simultaneous and intertwined reconstruction of both spectra and images through the direct application of a compressed sensing algorithm on the hyperspectral data cube. This approach differs from applying the algorithm separately to spectral and spatial dimensions, offering the potential for a more efficient hyperspectral imaging process.

## Materials and methods

### PbS CQDs synthesis

The PbS CQDs are synthesized using a revisited procedure of that described in ref. ^39^. Briefly, 0.45 g PbO, 2–20 g oleic acid, and 10 g 1-Octadecene (ODE) were loaded in a 100 mL flask and heated to 110 °C for 20 min under vacuum to obtain a clear solution. The temperature was adjusted to the desired injection temperature (95–185 °C) followed by a fast injection of 210 μL of (TMS)_2_S diluted in 5 mL of ODE. Immediately before injection, the heating mantle was removed and the solution cooled naturally after the injection. When the reaction solution reached 30 °C, 30 μL of raw solution was removed and dissolved in 2.5 mL of n-Hexane for absorption measurements. Before device fabrication, the PbS CQDs were purified twice using ethanol and redispersed in hexane.

### CQD filter fabrication

The glass substrate is soaked in isopropyl alcohol and ultrasonic cleaned for 30 min. After drying, the substrate is cleaned by plasma cleaner to improve the adhesion effect of CQDs on the surface of the substrate. Drop a solution of PbS CQDs (10% v v^−1^ octane in hexane) onto the center of the substrate and spread the solution. Cover the filter with a dust shield to prevent dust in the air from mixing with the material. After 24 h, the solvent can be completely volatilized to complete the deposition of the film. A black film can be observed on the substrate.

### Supplementary information

Supplemental Information
