# Supplementary material for: Quantum dot-enabled infrared hyperspectral imaging with single-pixel detection
Source: Light Sci Appl. 2024 May 28;13:121. doi: 10.1038/s41377-024-01476-4 (PMC11130170; doi:10.1038/s41377-024-01476-4)
Supplement: Supplementary file 1 — Supplemental Information [file 41377_2024_1476_MOESM1_ESM.docx]

**Supplementary Information for**

**Quantum dot-enabled infrared hyperspectral imaging with single-pixel detection**

**Authors**

Heyan Meng^1,#^, Yuan Gao^1,2,#,*^, Xuhong Wang^2^, Xianye Li^3^, Lili Wang^2^, Xian Zhao^2^& Baoqing Sun^1,2,*^

**Affiliations**

^1^ School of Information Sciences and Engineering, Shandong University, Qingdao 266237, China

^2^ Center for Optics Research and Engineering (CORE), Key Laboratory of Laser & Infrared System (Shandong University), Ministry of Education, Shandong University, Qingdao 266237, China

^3^ School of Mechanical, Electrical and Information Engineering, Shandong University, Weihai, 264209, China

^#^ These authors contributed equally to this work.

*Correspondence and requests should be addressed to Y.G. or to B.Q.S.

**Email addresses:**

*Yuan Gao ([yuan.gao@sdu.edu.cn](mailto:yuan.gao@sdu.edu.cn));
*Baoqing Sun ([baoqing.sun@sdu.edu.cn](mailto:baoqing.sun@sdu.edu.cn)).

**Note 1: Comparative analysis of signal-to-noise ratio in hyperspectral imaging using single-pixel detector vs. FPA.**

The single-pixel detector lacks spatial resolution. Conversely, FPA consist of a 2D detector array.

In single-pixel detection, image quality can be affected by fluctuations in illumination power and noise. This process can be mathematically expressed as:

Single-pixel detection:

$$\begin{aligned} O\left( A \right)=H^{T}\left( Y+B \right)\#\left( S1 \right) \end{aligned}$$

Here, *A* represents the reconstructed image, *H* is a known Hadamard matrix, *Y* is the sampled signal from the single-pixel detector, and *B* accounts for detector noise.

Conversely, FPA imaging is instantaneous and isn't affected by lighting power fluctuations. The influence of noise on FPA is distributed across each pixel, and the imaging process can be described as:

FPA imaging process:

$$\begin{aligned} O\left( A_{i,j} \right)=Y_{i,j}+B_{i,j}\#\left( S2 \right) \end{aligned}$$

where, $A_{i,j}$ is a certain pixel in the reconstructed image, $Y_{i,j}$ is the signal of the pixel corresponding to the single-pixel detector in FPA, and $B_{i,j}$ represents the detector noise of each single-pixel detector.

In linear photoelectric detection, dark noise is the predominant detection noise, independent of the illumination conditions. The statistical distribution of dark noise, denoted as *n_i_*, adheres to a Gaussian distribution $N(0,\sigma_{n}^{2})$. Various noise scenarios can be replicated by adjusting parameters within the noise model.

For the purpose of evaluating the noise tolerance of these two imaging methods, our experiment employed a 64 × 64 resolution image featuring the characters “SDU” as the imaging target, as depicted in Fig. S1. We introduced varying levels of noise during the acquisition of this target image. To simulate spectral reconstruction experiments, distinct spectral curves were applied to the three different letters, with each assigned a unique pseudocolor for clarity, as shown in Fig. S1.


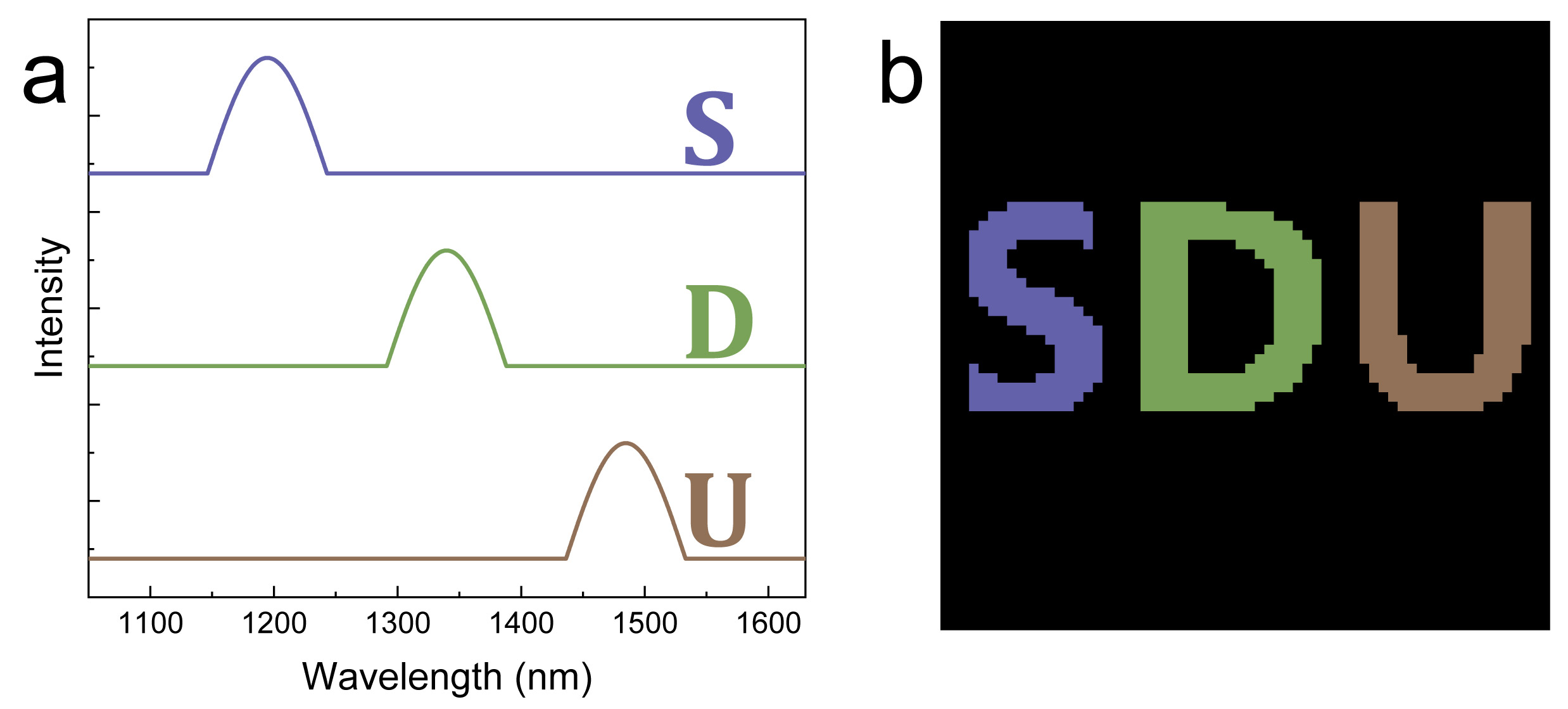


**Fig. S1 Simulation imaging experiment target.** **(a)** The spectral curves and pseudocolors of the different letters. **(b)** Experimental target after pseudocolors assignment.

In the experiment, images were captured using both the FPA and the single-pixel detector under ideal conditions. Subsequently, we employed 100 artificial transmission spectral curves to reconstruct spectral information at each pixel. The resulting three-dimensional data cubes with pseudocolor assignment are presented in Fig. S2 and S3.

Figure S2 displays the simulation outcomes of hyperspectral imaging for the letter “SDU” using the FPA. The top three rows of images in Fig. S2 correspond to the results obtained with the 1st, 50th, and 100th filters.

Throughout the experiment, noise affected the output signal of every detector in the FPA independently, leading to a reduction in image quality.


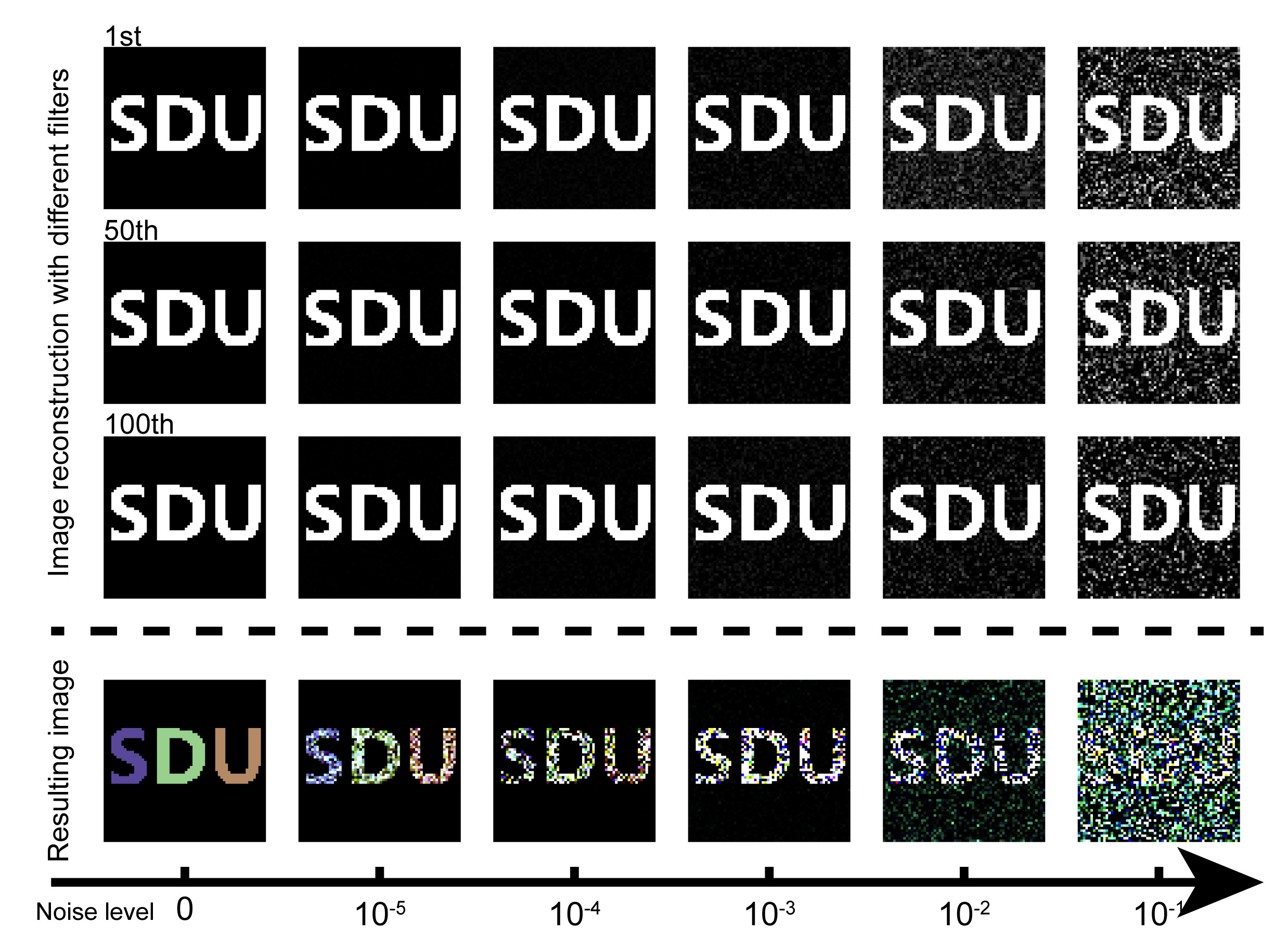


**Fig. S2 Reconstruction results under different noise levels using FPA.**

Figure S3 depicts the simulation outcomes of hyperspectral imaging for the letter “SDU” using a single-pixel detector. To facilitate a comparison, we have selected and normalized the images acquired with the 1st, 50th, and 100th filters to ensure they are within the same order of magnitude.

During the experiment, it’s essential to note that single-pixel detection differs from FPA-based detection, and the quality of the images is not directly affected by noise. Noise influences the measured values during the single-pixel detection process and indirectly impacts the imaging results.

Thanks to its remarkable noise tolerance, single-pixel detection can faithfully reconstruct the image of the target object even under conditions of high noise levels. As illustrated in Fig. S3, it's evident that single-pixel detection exhibits superior noise resistance compared to imaging with FPA.


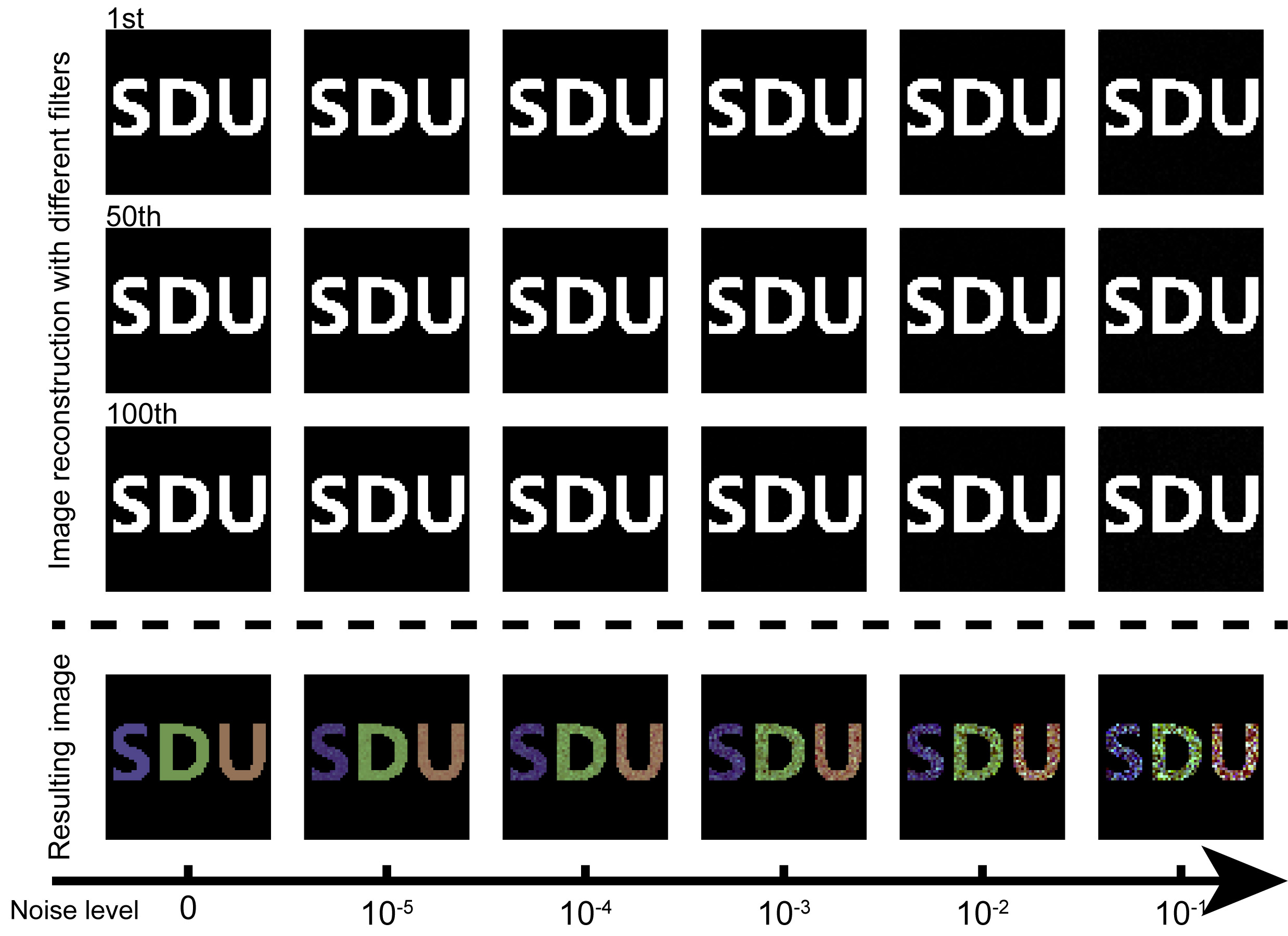


**Fig. S3 Reconstruction results under different noise levels using a single-pixel detector.**

To evaluate the quality of spectral reconstruction using FPA and single-pixel detector, we have presented the spectral reconstruction results for the letter “D” at different noise levels in Fig. S4.


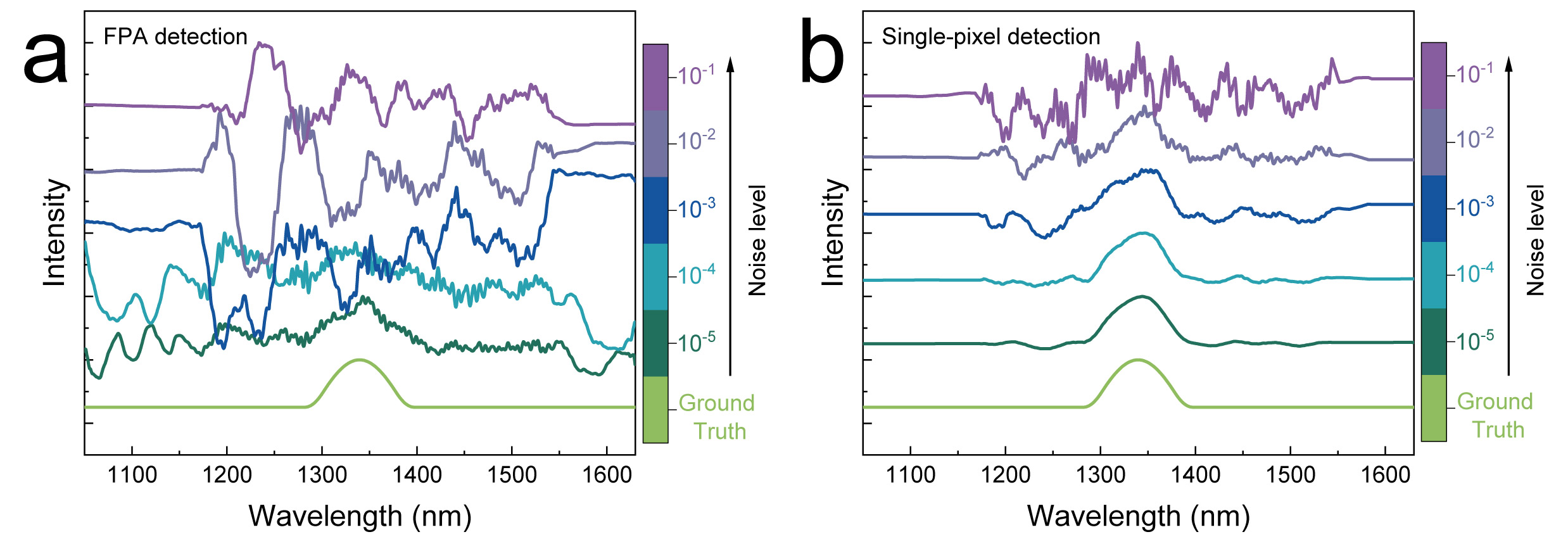


**Fig. S4 Comparison of spectral reconstruction results by (a) FPA and (b) single-pixel detection**

**Note 2: The effect of detector dark noise on imaging**

The impact of noise on the image reconstruction outcome is inherently tied to the chosen imaging method. In the case of FPA-based imaging, noise affects the output signal of each individual pixel in the array independently. This independent influence leads to a decrease in the overall image quality.

Under ideal conditions, each individual pixel detector within the array can accurately capture the spatial details of the target object. The output intensity value of each pixel in the array is denoted as $A_{ij}$, where $i, j=1, 2, 3\ldots n$, representing the location of the pixel on the array. When FPA are affected by noise, the acquired information is represented as $A_{ij}+B_{ij}$. The model is illustrated below:

$$\begin{aligned} \left[ \begin{matrix} \begin{matrix} A_{11} & A_{12} & A_{13} \\ A_{21} & A_{22} & A_{23} \\ A_{31} & A_{32} & A_{33} \end{matrix} & \cdots& \begin{matrix} A_{1n} \\ A_{21} \\ A_{31} \end{matrix} \\ \vdots& \ddots& \vdots\\ \begin{matrix} A_{n1} & A_{n2} & A_{n3} \end{matrix} & \cdots& A_{nn} \end{matrix} \right]\#\left( S3 \right) \end{aligned}$$

Therefore,

$$\begin{aligned} \left[ \begin{matrix} \begin{matrix} A_{11}+B_{11} & A_{12}+B_{12} & A_{13}+B_{13} \\ A_{21}+B_{21} & A_{22}+B_{22} & A_{23}+B_{23} \\ A_{31}+B_{31} & A_{32}+B_{32} & A_{33}+B_{33} \end{matrix} & \cdots& \begin{matrix} A_{1n}+B_{1n} \\ A_{21}+B_{21} \\ A_{31}+B_{31} \end{matrix} \\ \vdots& \ddots& \vdots\\ \begin{matrix} A_{n1}+B_{n1} & A_{n2}+B_{n2} & A_{n3} \end{matrix}+B_{n3} & \cdots& A_{nn}+B_{nn} \end{matrix} \right]\#\left( S4 \right) \end{aligned}$$

where $A_{ij}(i, j=1, 2, 3, \ldots, n^{2})$ is not only the intensity value of each pixel on the resulting image, but also the signal collected at the corresponding pixel of the array. $B_{ij}(i, j=1, 2, 3, \ldots, n^{2})$ is the dark noise introduced by different single-pixel detectors on the array.

Calculation the signal-to-noise ratio of the image obtained by FPA, as shown below:

$$\begin{aligned} {SNR}_{\mathrm{FPA}}=10\times\log_{10} \frac{\sum_{i=1}^{n} \left( \sum_{j=1}^{n} \left( A_{ij} \right) \right)}{\sum_{i=1}^{n} \left( \sum_{j=1}^{n} \left( B_{ij} \right) \right)}=10\times\log_{10} \frac{\sum_{k=1}^{n^{2}} \left( A_{k} \right)}{\sum_{k=1}^{n^{2}} \left( B_{k} \right)}\#\left( S5 \right) \end{aligned}$$

Likewise, during the process of single-pixel detection, perfect reconstruction of the target object can be achieved under ideal conditions. However, unlike FPA-based imaging, image quality isn't directly impacted by noise. Instead, noise affects the measured values in this process, indirectly influencing the overall imaging quality. The model is presented below:

$$\begin{aligned} \left[ \begin{matrix} \begin{matrix} H_{11} & H_{12} & H_{13} \\ H_{21} & H_{22} & H_{23} \\ H_{31} & H_{32} & H_{33} \end{matrix} & \cdots& \begin{matrix} H_{1n^{2}} \\ H_{2n^{2}} \\ H_{3n^{2}} \end{matrix} \\ \vdots& \ddots& \vdots\\ \begin{matrix} H_{n^{2}1} & H_{n^{2}2} & H_{n^{2}3} \end{matrix} & \cdots& H_{n^{2}n^{2}} \end{matrix} \right]\times\left[ \begin{matrix} \begin{matrix} A_{1} \\ A_{2} \\ A_{3} \end{matrix} \\ \begin{matrix} \vdots\\ A_{n^{2}} \end{matrix} \end{matrix} \right]=\left[ \begin{matrix} \begin{matrix} Y_{1} \\ Y_{2} \\ Y_{3} \end{matrix} \\ \begin{matrix} \vdots\\ Y_{n^{2}} \end{matrix} \end{matrix} \right]\#\left( S6 \right) \end{aligned}$$

Therefore,

$$\begin{aligned} \left[ \begin{matrix} \begin{matrix} H_{11} & H_{12} & H_{13} \\ H_{21} & H_{22} & H_{23} \\ H_{31} & H_{32} & H_{33} \end{matrix} & \cdots& \begin{matrix} H_{1n^{2}} \\ H_{2n^{2}} \\ H_{3n^{2}} \end{matrix} \\ \vdots& \ddots& \vdots\\ \begin{matrix} H_{n^{2}1} & H_{n^{2}2} & H_{n^{2}3} \end{matrix} & \cdots& H_{n^{2}n^{2}} \end{matrix} \right]\times\left[ \begin{matrix} \begin{matrix} A_{1} \\ A_{2} \\ A_{3} \end{matrix} \\ \begin{matrix} \vdots\\ A_{n^{2}} \end{matrix} \end{matrix} \right]=\left[ \begin{matrix} \begin{matrix} Y_{1}+B_{1} \\ Y_{2}+B_{2} \\ Y_{3}+B_{3} \end{matrix} \\ \begin{matrix} \vdots\\ Y_{n^{2}}+B_{n^{2}} \end{matrix} \end{matrix} \right]\#\left( S7 \right) \end{aligned}$$

where, $H_{ij}(i, j=1, 2, 3, \ldots, n^{2})$ is the modulation matrix in the single-pixel detection process. In order to achieve high quality image reconstruction, Hadamard matrix is used as modulation matrix in the experiment. $A_{k}(k=1, 2, 3, \ldots, n^{2})$ is the intensity value of each pixel on the resulting image by its spatial position. $Y_{k}(k=1, 2, 3, \ldots, n^{2})$ is the measured value in the single-pixel detection process. $B_{k}(k=1, 2, 3, \ldots, n^{2})$ is the detector dark noise.

Next, ideally analyze the measured value $Y_{k}$ and the resulting image $A_{k}$. Multiply both sides of this equation by the inverse of the Hadamard matrix. By virtue of the fact that the inverse of the Hadamard matrix is the same as itself, the Eq. S7 can be rewritten as Eq. S8.

$$\begin{aligned} \left[ \begin{matrix} \begin{matrix} H_{11} & H_{12} & H_{13} \\ H_{21} & H_{22} & H_{23} \\ H_{31} & H_{32} & H_{33} \end{matrix} & \cdots& \begin{matrix} H_{1n^{2}} \\ H_{2n^{2}} \\ H_{3n^{2}} \end{matrix} \\ \vdots& \ddots& \vdots\\ \begin{matrix} H_{n^{2}1} & H_{n^{2}2} & H_{n^{2}3} \end{matrix} & \cdots& H_{n^{2}n^{2}} \end{matrix} \right]\times\left[ \begin{matrix} \begin{matrix} H_{11} & H_{12} & H_{13} \\ H_{21} & H_{22} & H_{23} \\ H_{31} & H_{32} & H_{33} \end{matrix} & \cdots& \begin{matrix} H_{1n^{2}} \\ H_{2n^{2}} \\ H_{3n^{2}} \end{matrix} \\ \vdots& \ddots& \vdots\\ \begin{matrix} H_{n^{2}1} & H_{n^{2}2} & H_{n^{2}3} \end{matrix} & \cdots& H_{n^{2}n^{2}} \end{matrix} \right]\times\left[ \begin{matrix} \begin{matrix} A_{1} \\ A_{2} \\ A_{3} \end{matrix} \\ \begin{matrix} \vdots\\ A_{n^{2}} \end{matrix} \end{matrix} \right] \\ =\left[ \begin{matrix} \begin{matrix} H_{11} & H_{12} & H_{13} \\ H_{21} & H_{22} & H_{23} \\ H_{31} & H_{32} & H_{33} \end{matrix} & \cdots& \begin{matrix} H_{1n^{2}} \\ H_{2n^{2}} \\ H_{3n^{2}} \end{matrix} \\ \vdots& \ddots& \vdots\\ \begin{matrix} H_{n^{2}1} & H_{n^{2}2} & H_{n^{2}3} \end{matrix} & \cdots& H_{n^{2}n^{2}} \end{matrix} \right]\times\left[ \begin{matrix} \begin{matrix} Y_{1} \\ Y_{2} \\ Y_{3} \end{matrix} \\ \begin{matrix} \vdots\\ Y_{n^{2}} \end{matrix} \end{matrix} \right]\#\left( S8 \right) \end{aligned}$$

Thus:

$$n^{2}\times\left[ \begin{matrix} \begin{matrix} A_{1} \\ A_{2} \\ A_{3} \end{matrix} \\ \begin{matrix} \vdots\\ A_{n^{2}} \end{matrix} \end{matrix} \right]=\left[ \begin{matrix} \begin{matrix} H_{11} & H_{12} & H_{13} \\ H_{21} & H_{22} & H_{23} \\ H_{31} & H_{32} & H_{33} \end{matrix} & \cdots& \begin{matrix} H_{1n^{2}} \\ H_{2n^{2}} \\ H_{3n^{2}} \end{matrix} \\ \vdots& \ddots& \vdots\\ \begin{matrix} H_{n^{2}1} & H_{n^{2}2} & H_{n^{2}3} \end{matrix} & \cdots& H_{n^{2}n^{2}} \end{matrix} \right]\times\left[ \begin{matrix} \begin{matrix} Y_{1} \\ Y_{2} \\ Y_{3} \end{matrix} \\ \begin{matrix} \vdots\\ Y_{n^{2}} \end{matrix} \end{matrix} \right]$$

$$\begin{aligned} =\left[ \begin{matrix} \begin{matrix} H_{11}\times Y_{1}+H_{12}\times Y_{2}+H_{13}\times Y_{3}+\cdots+H_{1n^{2}}\times Y_{n^{2}} \\ H_{21}\times Y_{1}+H_{22}\times Y_{2}+H_{23}\times Y_{3}+\cdots+H_{2n^{2}}\times Y_{n^{2}} \\ H_{31}\times Y_{1}+H_{32}\times Y_{2}+H_{33}\times Y_{3}+\cdots+H_{3n^{2}}\times Y_{n^{2}} \end{matrix} \\ \begin{matrix} \vdots\\ H_{n^{2}1}\times Y_{1}+H_{n^{2}2}\times Y_{2}+H_{n^{2}3}\times Y_{3}+\cdots+H_{n^{2}n^{2}}\times Y_{n^{2}} \end{matrix} \end{matrix} \right]\#\left( S9 \right) \end{aligned}$$

Each pixel in the reconstructed image can be represented by the following formula:

$$\left[ \begin{matrix} \begin{matrix} A_{1} \\ A_{2} \\ A_{3} \end{matrix} \\ \begin{matrix} \vdots\\ A_{n2} \end{matrix} \end{matrix} \right]=\frac{1}{n^{2}}\left[ \begin{matrix} \begin{matrix} H_{11}\times Y_{1}+H_{12}\times Y_{2}+H_{13}\times Y_{3}+\cdots+H_{1n^{2}}\times Y_{n^{2}} \\ H_{21}\times Y_{1}+H_{22}\times Y_{2}+H_{23}\times Y_{3}+\cdots+H_{2n^{2}}\times Y_{n^{2}} \\ H_{31}\times Y_{1}+H_{32}\times Y_{2}+H_{33}\times Y_{3}+\cdots+H_{3n^{2}}\times Y_{n^{2}} \end{matrix} \\ \begin{matrix} \vdots\\ H_{n^{2}1}\times Y_{1}+H_{n^{2}2}\times Y_{2}+H_{n^{2}3}\times Y_{3}+\cdots+H_{n^{2}n^{2}}\times Y_{n^{2}} \end{matrix} \end{matrix} \right]$$

$$\begin{aligned} =\frac{1}{n^{2}}\left[ \begin{matrix} \begin{matrix} \sum_{l=1}^{n^{2}} \left( H_{1l}\times Y_{l} \right) \\ \sum_{l=1}^{n^{2}} \left( H_{2l}\times Y_{l} \right) \\ \sum_{l=1}^{n^{2}} \left( H_{3l}\times Y_{l} \right) \end{matrix} \\ \begin{matrix} \vdots\\ \sum_{l=1}^{n^{2}} \left( H_{n^{2}l}\times Y_{l} \right) \end{matrix} \end{matrix} \right]\#\left( S10 \right) \end{aligned}$$

Thus:

$$\begin{aligned} A_{k}=\frac{1}{n^{2}}\times\sum_{l=1}^{n^{2}} \left( H_{kl}\times Y_{l} \right)\#\left( S11 \right) \end{aligned}$$

As described above, the influence of noise on single-pixel detection is to indirectly affect the imaging results by affecting the measured values in the correlation calculation. Therefore, under noisy conditions, the Eq. S11 can be written as:

$$\begin{aligned} A_{k}^{'}=\frac{1}{n^{2}}\times\sum_{l=1}^{n^{2}} \left( H_{kl}\times\left( Y_{l}+B_{l} \right) \right)=A_{k}+\frac{1}{n^{2}}\times\sum_{l=1}^{n^{2}} \left( {H_{kl}\times B}_{l} \right)\# \left( S12 \right) \end{aligned}$$

Calculate the signal-to-noise ratio of the image obtained by a single-pixel detector, as shown below:

$$\begin{aligned} {SNR}_{\mathrm{SPD}}=10\times\log_{10} \frac{\sum_{k=1}^{n^{2}} \left( A_{k}^{2} \right)}{\sum_{k=1}^{n^{2}} \left( \frac{1}{n^{2}}\times\sum_{l=1}^{n^{2}} \left( {H_{kl}\times B}_{l} \right) \right)^{2}}\# \left( S13 \right) \end{aligned}$$

There are only 1 and -1 in the Hadamard matrix. Therefore, in the calculation process of single-pixel detection, the elements $H_{kl}(k, l=1, 2, 3, \ldots, n^{2})$ is not always equal to 1, so:

$$\begin{aligned} \sum_{k=1}^{n^{2}} \left( \frac{1}{n^{2}}\times\sum_{l=1}^{n^{2}} \left( {H_{kl}\times B}_{l} \right) \right)^{2}<\sum_{k=1}^{n^{2}} \left( \frac{1}{n^{2}}\times\sum_{l=1}^{n^{2}} \left( {1\times B}_{l} \right) \right)^{2}\#\left( S14 \right) \end{aligned}$$

In addition, due to the non-negative nature of the detector dark noise, the above formula can be written as:

$\begin{aligned} \sum_{k=1}^{n^{2}} \left( \frac{1}{n^{2}}\times\sum_{l=1}^{n^{2}} \left( {1\times B}_{l} \right) \right)^{2}<\sum_{k=1}^{n^{2}} \left( B_{k} \right)^{2}\#\left( S15 \right) \end{aligned}$

So, the following relationship exists:

$$\begin{aligned} \sum_{k=1}^{n^{2}} \left( \frac{1}{n^{2}}\times\sum_{l=1}^{n^{2}} \left( {H_{kl}\times B}_{l} \right) \right)^{2}<\sum_{k=1}^{n^{2}} \left( B_{k} \right)^{2}\#\left( S16 \right) \end{aligned}$$

Thus:

$${SNR}_{\mathrm{SPD}}=10\times\log_{10} \frac{\sum_{k=1}^{n^{2}} \left( A_{k}^{2} \right)}{\sum_{k=1}^{n^{2}} \left( \frac{1}{n^{2}}\times\sum_{l=1}^{n^{2}} \left( {H_{kl}\times B}_{l} \right) \right)^{2}}$$

$\begin{aligned} >10\times\log_{10} \frac{\sum_{k=1}^{n^{2}} \left( A_{k} \right)}{\sum_{k=1}^{n^{2}} \left( B_{k} \right)}{=SNR}_{\mathrm{FPA}}\#\left( S17 \right) \end{aligned}$

As shown in Eq. S17, single-pixel detection has a higher noise tolerance than FPA detection at the same noise level, which is graphically illustrated in Fig. S2 and S3.

**Note 3: Hyperspectral image reconstruction algorithm**

We define the target as a matrix of order of $L$ by $K$, where $L$ is the number of spectral data points per for each pixel and $K$ is the number of pixels in the target. So, we can define the transmitted spectra on color filters as a matrix of order of $m$ by $L$, where $m$ is the number of color filters. Similarly, we can define the patterns on DMD as a matrix of order of $K$ by $n$, where $n$ is the number of patterns. As a result, Eq. 1 can be written as:

$$\begin{aligned} Y=FTH\#\left( S18 \right) \end{aligned}$$

where $Y$ (a matrix of order of $m$ by $n$) representing the output signal of the single-pixel detector.

In the experiment, we performed a single-pixel detection each time we added a color filter to the system. Then, we are able to obtain a series of single-pixel images whose number is equal to the number of color filters. This process is mathematically expressed as a matrix operation on the Eq. S18 as shown above.

Since the target was fully sampled, the number of patterns was the same as the number of pixels, $n$ was equal to $K$. The property of a Hadamard matrix is that it is orthogonal and its transpose is equal to itself. Therefore, the product of two Hadamard matrices is a constant. The Multiply both sides of the equation by $H$ to the right. Eq. S18 can be written as:

$$\begin{aligned} YH=FTHH=FT*K\#\left( S19 \right) \end{aligned}$$

Thus,

$$\begin{aligned} Y^{'}=FT\#\left( S20 \right) \end{aligned}$$

where $Y^{'}$ (a matrix of order of $m$ by $K$) representing the single-pixel detection results under spectral modulation. Each row of this matrix is an individual single-pixel image.

After that, we can solve the spectral curve of each pixel through these images. For the spectrum of a pixel in the object, the corresponding column of $Y^{'}$ is the intensity value under the modulation of different filters. This process is mathematically expressed as a matrix operation on the Eq. S20 as shown above.

Keep $F$ stable and extract column vectors of the same position to a new equation:

$$\begin{aligned} Y_{k}^{'}=FT_{k}\#\left( S21 \right) \end{aligned}$$

We adapted the compressive-sensing–based Gap-TV algorithm for spectral reconstruction, which has been demonstrated to effectively recover latent signals from a small number of measurements.

After the spectrum of all the pixels are obtained, they can be reassembled to reconstruct a 3D data cube. This is the result of our reconstruction.

**Note 4: Performance analysis of three kinds of filters**

In the experiment, we employed 50 filters with three distinct transmissive characteristics to encode and reconstruct two Gaussian peaks positioned apart from each other (the first peak of the Gaussian target curve located at 1300 nm, while the separation distance between the two peaks varied). Both peaks exhibited a FWHM of 1 nm, with the separation distance denoted as D. The transmission spectra of the three types of filters used in the experiment are illustrated in Fig. S5.

Figure S6a, d, and g present the spectral reconstruction outcomes using CQD filters, while Fig. S6b, e, and h depict the results obtained with long-pass filters, and Fig. S6c, f, and i showcase the results obtained with band-pass filters. It was noted that when the Gaussian target peaks were separated by 7 nm, the utilization of CQD filters enabled the differentiation between these two target peaks, whereas long-pass filters and band-pass filters failed to do so. Only when the separation between the two Gaussian target peaks reached 16 nm were long-pass filters and band-pass filters able to achieve distinguishability. Drawing from the experimental findings and considering that CQD filters demonstrate greater variability in spectral transmissive characteristics compared to long-pass and band-pass filters, it can be concluded that utilizing CQD filters for spectral encoding enables more effective modulation of target spectra across a range of wavelengths, consequently resulting in enhanced spectral resolution.


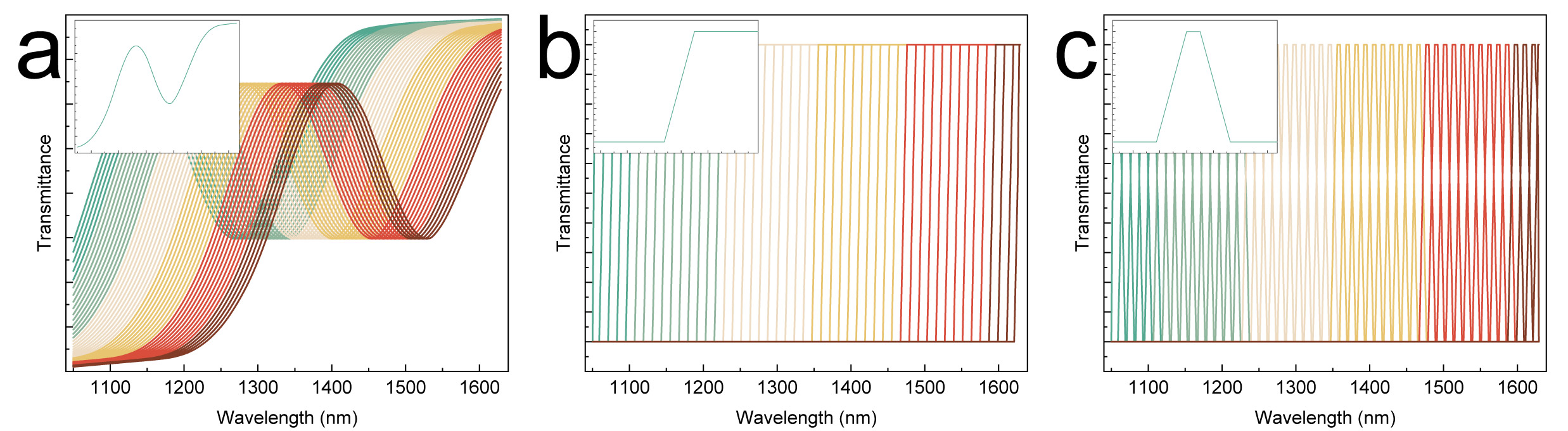


**Fig. S5** The simulated transmission spectra of **(a)** CQD filters, **(b)** long-pass filters, and **(c)** band-pass filters, respectively.


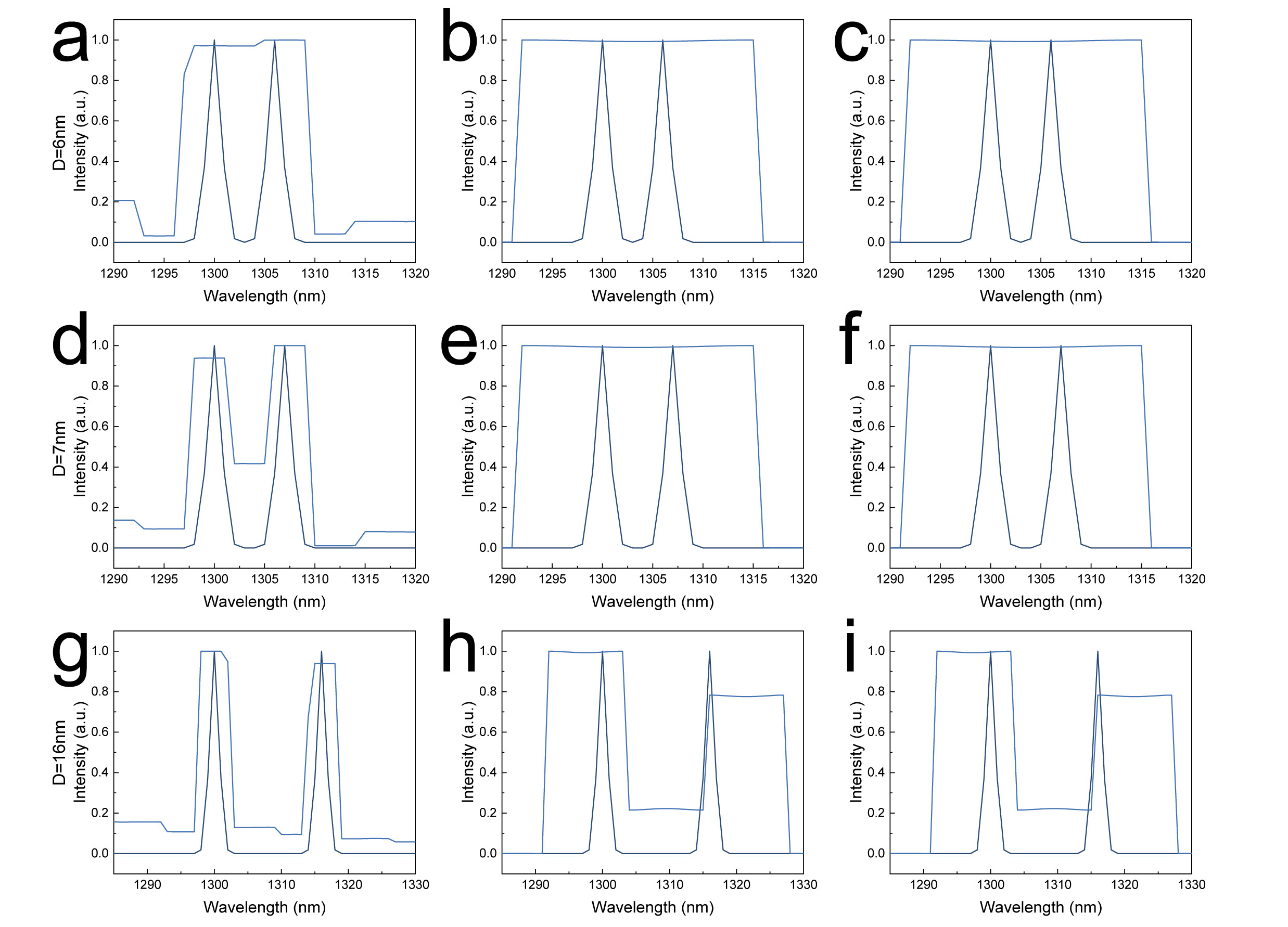


**Fig. S6** The simulated spectral reconstruction using **(a, d, and g)** CQD filter, **(b, e, and h)** long-pass filters, and **(c, f, and i)** band-pass filters.

**Note 5: The relationship between system performance and the quantity of CQD filters**

To experimentally examine the relationship between system performance and the quantity of CQD filters utilized, we conducted a series of experiments employing varying numbers of these filters (see Fig. S7a and b). In these experiments, we employed a Gaussian curve with a central wavelength of 1300 nm and a FWHM of 1 nm as the target (see Fig. S7c and d). This target curve was then reconstructed using different quantities of filters: 10, 20, 30, 40, and 50.

The spectral reconstruction outcomes are illustrated in Fig. S8a and b. Employing only ten filters resulted in a reconstructed peak with a FWHM of 70.72 nm, which is 71 times wider than the actual width. Conversely, as the number of filters increased, the FWHM of the reconstructed peak decreased from 70.72 nm to 8.24 nm (as shown in Fig. S8 c). As a result, increasing the number of filters resulted in improved accuracy in spectral reconstruction and enhanced spectral resolution, aligning with our simulation findings depicted in Fig. 4a of the main text.


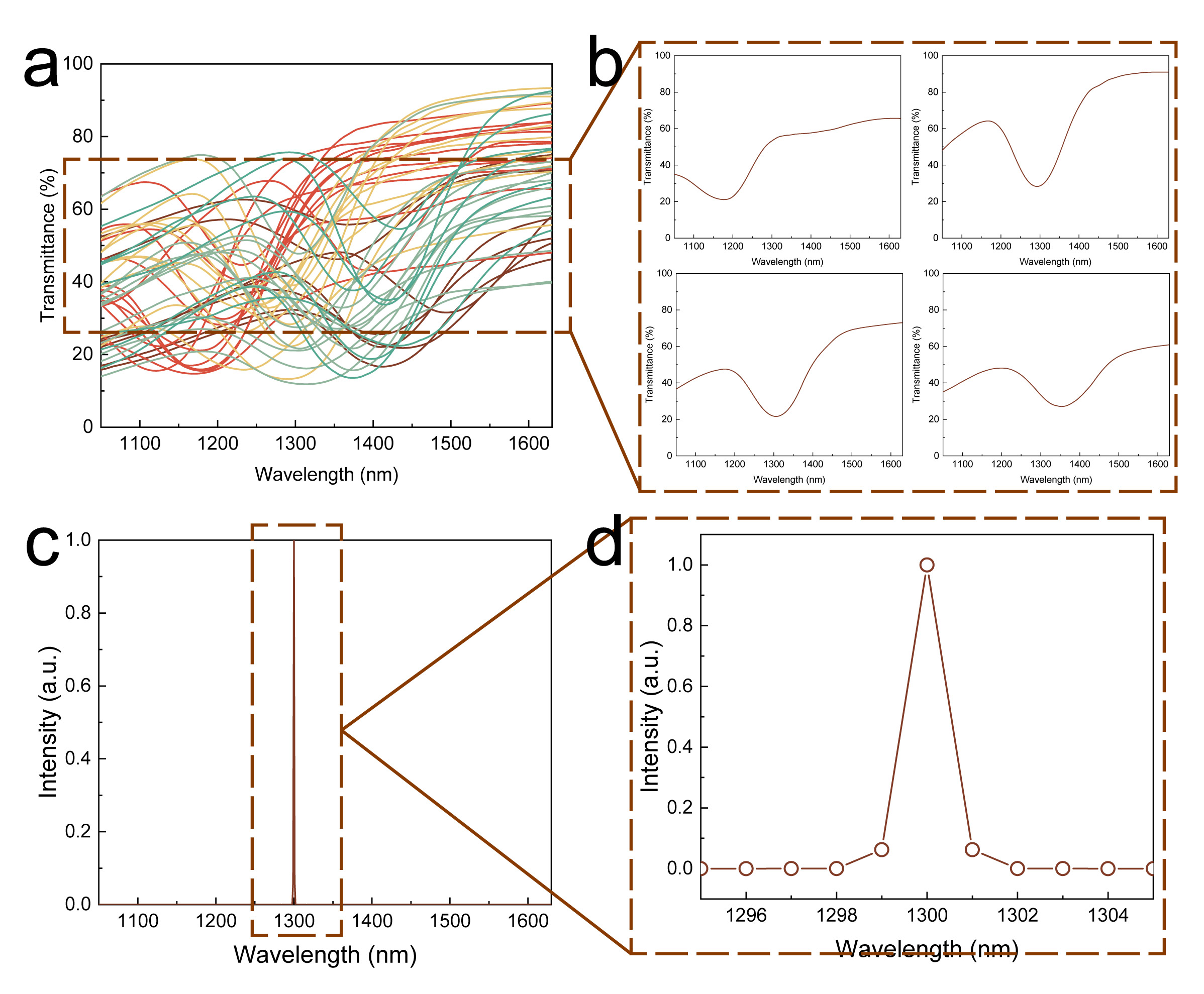


**Fig. S7 (a)** and **(b)** display the measured transmission spectra of the CQD filters, while **(c)** and **(d)** represent a Gaussian peak centered at 1300 nm as the target.


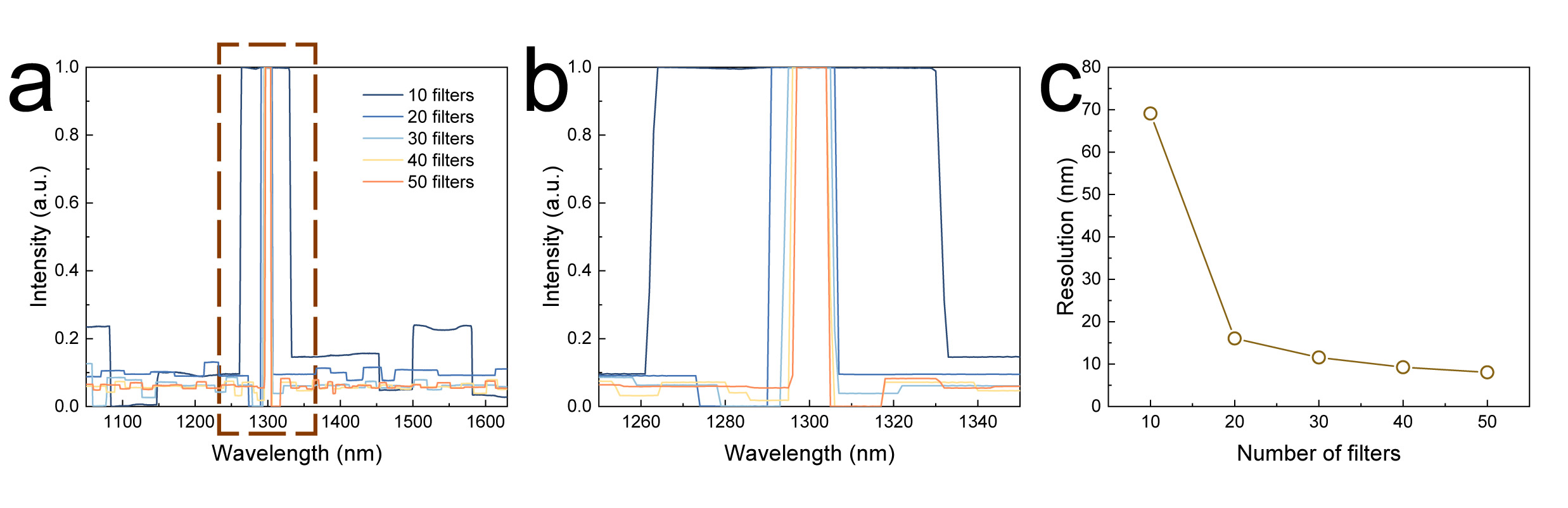


**Fig. S8 (a)** The reconstructed peaks using an increasing number of CQD filters. **(b)** A close-up view of the reconstructed peaks. **(c)** The widths of the reconstructed peaks as the number of CQD filters increases.

**Note 6: Correlation of the spectrum and pseudocolor**

The CIE 1931 color space establishes precise connections between electromagnetic spectrum wavelengths and the colors perceived by the human visual system. It encompasses the entire range of colors visible to an average human with normal vision. Within this color space, each primary color (red, green, and blue) is associated with a color-matching function [$\overline{x}(\lambda)$, $\overline{y}(\lambda)$, and $\overline{z}(\lambda)$]. These functions can be thought of as the spectral sensitivity curves of three linear light detectors that produce the CIE tristimulus values X, Y, and Z. Together, these functions collectively define the CIE standard observer.

Building on this theoretical framework, we utilize colors within the visible spectrum to represent the infrared spectral information of our target. The infrared spectral curve is effectively mapped into the CIE color space. By applying stretching and reassembly operations along the wavelength scale, it becomes aligned with the color matching function. Through a mathematical process involving the multiplication and summation of the spectral curve and the matching function, we calculate the weight of the curve on the *x*, *y*, and *z* components. This weight is then associated with three primary colors (red, green, and blue) to derive the pseudocolor representation of the spectral curve.


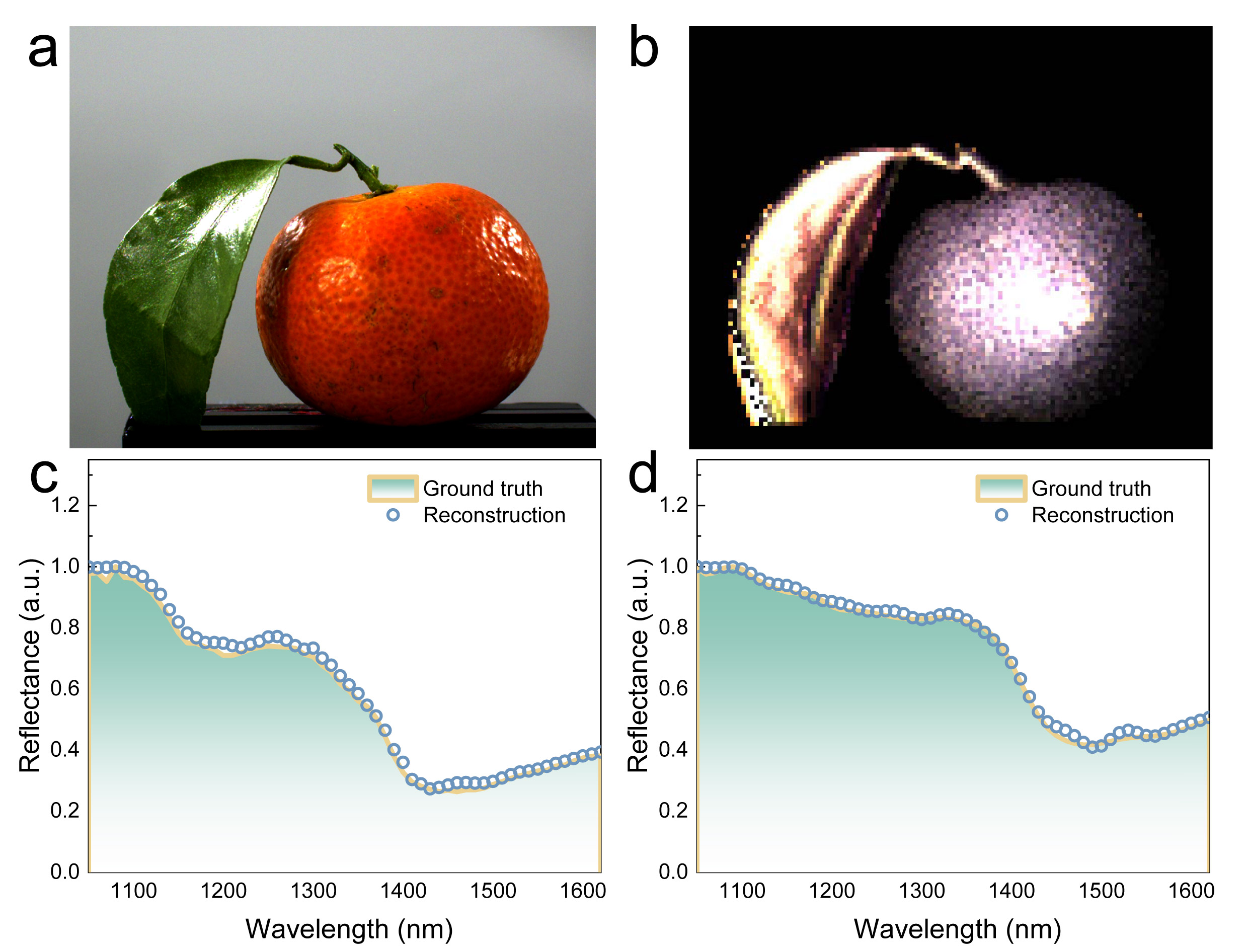


**Fig. S9 Hyperspectral image of an orange. (a)** Color photo and **(b)** hyperspectral image of an orange. The reconstructed reflection spectra of the **(c)** peel and **(d)** leaf of the orange.
